# Serum chemistry and electrolyte alterations in sled dogs before and after a 1600 km race: dietary sodium and hyponatraemia*

**DOI:** 10.1017/jns.2014.39

**Published:** 2014-09-25

**Authors:** Valentina Ermon, Molly Yazwinski, Justin G. Milizio, Joseph J. Wakshlag

**Affiliations:** Department of Clinical Sciences, Cornell University College of Veterinary Medicine, Veterinary Medical Center 1-120, Box 34, Ithaca, NY 14850, USA

**Keywords:** Sodium, Potassium, Sled dogs, Serum chemistry

## Abstract

Sled dogs are known to develop numerous serum biochemical changes due to endurance exercise. Previous studies have suggested that mild hyponatraemia and hypokalaemia can develop during endurance racing. The aim of the present study was to determine if serum biochemical alterations are similar to previous reports, and if electrolyte alterations are still present with present feeding practices utilised by mushers. Serum chemistries were obtained from 26 Alaskan Huskies belonging to 3 different teams, before and after a 1600 km race. Meals and snacks were analysed via calculation to determine daily macronutrient and electrolyte intake. Numerous biochemical alterations were observed including significant differences in serum total protein, albumin, globulin, aspartate aminotransferase, creatine kinase, TAG, NEFA and urea nitrogen (*P* < 0·05). Serum electrolyte status revealed a mild, yet significant decrease in serum sodium (*P* = 0·002); and serum potassium was not significantly different (*P* = 0·566). Further examination of the sodium intake across the three teams revealed two teams with an average daily intake of approximately 8·5 g/dog/d (700 mg/4184 kJ) and the other team consuming 11·1 g/dog/d (1200 mg/4184 kJ). Regression analysis shows a significant modest positive correlation between serum sodium decrease and sodium intake per metabolic body weight of the dogs, as well as a modest positive correlation between sodium intake and serum potassium implicating the renin–angiotensin aldosterone system as a major factor involved in sodium and potassium homoeostasis. These findings suggest that consumption of approximately 0·9 g/kg^0·75^ (1·2 g/4184 kJ) of sodium per d may prevent exercise-induced decreases in sodium and potassium.

## Introduction

Sled dogs competing in long-distance races are the ultimate model of endurance athletes. Previous studies have shown electrolyte changes^(^^1^^–^^4^^)^, increase in markers of muscle damage^(^^1^^,^^2^^,^^5^^,^^6^^)^, decrease in serum proteins concentrations^(^^1^^–^^4^^)^ and a high-energy consumption with extreme aerobic capacity^(^^1^^,^^7^^,^^8^^)^. Electrolyte alterations in endurance sled dogs have shown mild post-race hyponatraemia and hypokalaemia^(^^2^^–^^4^^)^, although most dogs usually remain within the reference range for these two electrolytes. Similar electrolyte changes can be found after endurance exercise in human subsjects^(^^9^^–^^11^^)^ and horses^(^^12^^,^^13^^)^; which are attributed to electrolyte loss in sweat and excess of free water intake. However, in dogs heat load is dissipated only via convection and radiation during respiration (panting) and not through sweating^(^^14^^)^. Therefore the mechanisms of exercise-related hyponatraemia and hypokalaemia shown in past studies in sled dogs competing in long-distance races is not completely understood, but appear to be due to excessive protein catabolism (urea generation), increased water turnover and modest deficiency in sodium intake^(^^3^^,^^4^^)^. In an effort to conserve sodium endurance dogs appear to activate the renin–aldosterone system which contributes to potassium losses and eventual hypokalaemia due to depletion of body reservoirs and increased renal losses^(^^3^^,^^4^^,^^15^^)^. However, a recent study by McKenzie *et al.* examined serum chemistry alteration in sled dogs and showed a tendency of sodium to increase instead of decrease with a more mild yet significant decrease in potassium^(^^5^^)^.

The role of dietary intake of sodium on this phenomenon in endurance sled dogs has not been investigated^(^^3^^)^. The aim of the present study is to determine if current diets based on commercial dog food and meat during racing are sufficient for sled dogs to maintain the serum sodium and potassium. To test this hypothesis sodium intake was examined across three teams with one team adding supplemental salt to the diet in an attempt to increase intake by approximately 1 g/dog/d (one tablespoon of table salt equating to 5·2 g of sodium per meal fed each day; average number of meals being 3 per d; total of 15·6 g to fourteen dogs); and to examine the pre- and post-racing sodium and potassium changes across dogs to look for correlations with calculated presumed intake per kg metabolic body weight across all dogs.

## Experimental design

All procedures used in the present study were approved by the Cornell University Institutional Animal Care and Use Committee and the Yukon Quest Board of Directors Ethics and Animal Use Committee. Four teams of Alaskan Sled dogs were recruited during the 2011 Yukon Quest race. Of the four teams, only three teams finished the race, and twenty-six dogs of the original fifty-four completed the race having both pre- and post-blood samples acquired. The genders consisted of fourteen males and twelve females and they ranged from 2 to 8 years old. The average weight was 23·4 (3·4) kg. All dogs had a physical examination and weights taken by a race veterinarian before the race start (Whitehorse, Yukon Territory, Canada), at the midpoint of the race (Dawson City, Yukon Territory, Canada), and end of the race (Fairbanks, Alaska, USA). All dogs were deemed healthy at each point.

### Blood sample collection

A blood sample (5 ml) was collected by a cephalic venepuncture from each dog 72 h before the starting of the race and within 2–4 h after the end of the race. All dogs had fasted minimally 6 h before blood draw up to 18 h. It was placed in a 7 ml red top plastic coagulation tube and centrifuged at 4000 ***g*** for 5 min within 30 min of collection. Serum was collected and transferred to cryovials and immediately frozen at −20°C and shipped to the laboratory on dry ice. Serum samples were stored at −80°C for 4 months until they were analysed. Serum concentration of sodium, potassium, chloride, bicarbonate, anion gap, creatinine, urea, total protein, globulin, albumin, cholesterol, TAG, glucose, creatine kinase, alanine-aminotransferase, aspartate-aminotransferase and NEFA were measured at the Cornell University Diagnostic Laboratory using the Hitachi 911 Chemistry Analyser.

### Team and diet analysis

Based on musher questionnaire and weighed food at Dawson check point the frequency and amount of commercial dog food used in meals and watering was assessed with energy and substrate content being reported elsewhere^(^^16^^)^. Team one supplied approximately 5·2 g of sodium from table salt (one tablespoon) to each meal (three per d) for the first 8 of total 10 d of racing. The salt supplementation was discontinued due to the musher running out of salt after day 8. Data on metabolisable energy, crude protein, crude fat, nitrogen-free extract, crude fibre, major mineral content of commercial dog foods used by the three mushers was acquired from the companies (Red Paw Poweredge 26K, Redpaw; Dr Tim's Dog Food, Momentum; Caribou Creek Dog Food, Caribou Creek Gold). All meats and relative amounts (kg) used and frequency of use in meals, water and snacks was assessed based on musher response during the race. The meats samples were analysed (Dairy One, Ithaca, NY) to assess crude protein, crude fat, crude fibre, dietary ash, sodium, potassium, magnesium, copper, iron, zinc and manganese. The data were compiled utilising an excel spreadsheet into daily consumption of sodium and potassium based on diet history during the race. Since the numbers of dog varied across the race for each team, the mean number of dogs during the entire race was utilised as an assumed dietary intake of sodium or potassium/dog/d for further data analysis. The average daily intake of sodium and potassium per dog was assessed per kg metabolic body weight based on the weight collected at Dawson Checkpoint (midpoint) and was compared to the mEq changes in serum potassium and sodium from pre- to post-race.

### Statistical analysis

All statistical testing was performed using SigmaPlot 11.0 (Systat Software, San Jose, CA). Shapiro–Wilks test was used to verify the normal distribution of values and since nearly all values conformed to normalcy a paired Student's *t* test was used to compare pre- and post-race values in the serum chemistry. Student's *t* test was also used to compare the pre- and post-race values of sodium for each team individually. Linear regression analysis was performed to assess the correlation between sodium intake per kg metabolic body weight and change in serum sodium and potassium status, as well as correlation between sodium intake and changes in serum potassium intake to establish whether sodium intake was related to the decrease in serum potassium concentrations. *P* values of <0·05 were considered significant for all tests performed.

## Results

Changes in serum electrolyte concentrations between pre- and post-race across all dogs are shown in Table 1. There was only a mild yet significant decrease in sodium from 146·9 (1·6) mEq/l to 145·1 (2·1) mEq/l (*P* = 0·002), but it remained within the normal ranges. Post-race potassium concentrations was not significantly lower than pre-race value, with a mean decreasing only from 4·4 (0·3) mEq/l to 4·3 (0·3) mEq/l (*P* = 0·566); and remained within normal range. Serum urea increased significantly (*P* < 0·001) (Table 1). Serum values of total protein, albumin and globulin decreased significantly (*P* < 0·001, *P* < 0·001 and *P* = 0·003, respectively) (Table 1). Significant increases in creatine kinase (*P* < 0·001), alanine aminotransferase (*P* < 0·001) and aspartate aminotransferase (*P* < 0·001; Table 1) were observed.

**Table 1.** Serum chemistry values (means and standard deviations) of twenty-six dogs before and after a 1600 km race

	Pre-race	Post-race	Reference range	*P*
Sodium (mEq/l)*	146·9 (1·6)	145·1 (2·1)	144–153	0·002
Potassium (mEq/l)	4·4 (0·3)	4·3 (0·3)	3·7–5·4	0·566
Chloride (mEq/l)	110·8 (1·7)	110·3 (1·8)	114–122	0·297
Bicarbonate (mEq/l)	21·8 (1·8)	20·8 (2·5)	17–24	0·09
Anion gap (mEq/l)	18·7 (1·5)	18·0 (2·3)	15–25	0·159
Urea (mg/dl)	23·2 (5·8)	46·7 (6·0)	6–22	<0·001
Creatinine (mg/dl)	0·7 (0·2)	0·8 (0·1)	0·4–1·5	0·432
Total protein (g/dl)	6·1 (0·3)	5·3 (0·4)	5·5–7·1	<0·001
Albumin (g/dl)	3·9 (0·2)	3·2 (0·3)	2·3–3·1	<0·001
Globulin (g/dl)	2·2 (0·2)	2·1 (0·2)	2·7–4·4	0·003
Glucose (mg/dl)	97·3 (13·6)	88·6 (0·2)	17–43	0·001
AST (U/l)	29·3 (8·3)	64·1 (19·6)	15–43	<0·001
ALT (U/l)	50·9 (12·7)	79·0 + 19·6	20–98	<0·001
Cholesterol (mg/dl)	239·5 (52·9)	241·6 (40·6)	229–349	0·777
TAG (mg/dl)	46·0 (14·4)	55·2 (17·0)	14–54	0·012
Creatine kinase (U/l)	149·3 (72·9)	402·4 (234·1)	31–217	<0·001
BHBA (µmol/l)	0·1 (0·1)	0·1 (0·1)	NE	0·78
NEFA	1·1 (0·4)	0·5 (0·2)	NE	<0·001

AST, apartate-aminotrasferase; BHBA, β-hydroxybutyrate; NE, not established. Values are means (sd). Pre-race and post-race: values measured before and after the race.

*Indicates that eight dogs of the twenty-six total dogs were receiving sodium chloride supplementation to their diet.

Average daily sodium intake for each dog was higher for team 1. This team was supplementing salt to the diet with a total sodium intake of approximately 11·1 g/d per dog (1·21 g/4184 kJ). This sodium intake was higher than the other two teams using dog food and meat as the primary sources of dietary sodium (team 2 = 8·7 g/d per dog (0·72 g/4184 kJ) team 3 = 8·3 g/d per dog (0·69 g/4184 kJ)). The daily potassium intakes calculated for the three teams were: team 1 = 13·3 g/dog/d (1·45 g/4184 kJ), team 2 = 17·7 g/dog/d1 (1·44 g/4184 kJ) and team three = 17·5 g/dog/d (1·46 g/4184 kJ).

**Fig. 1.**
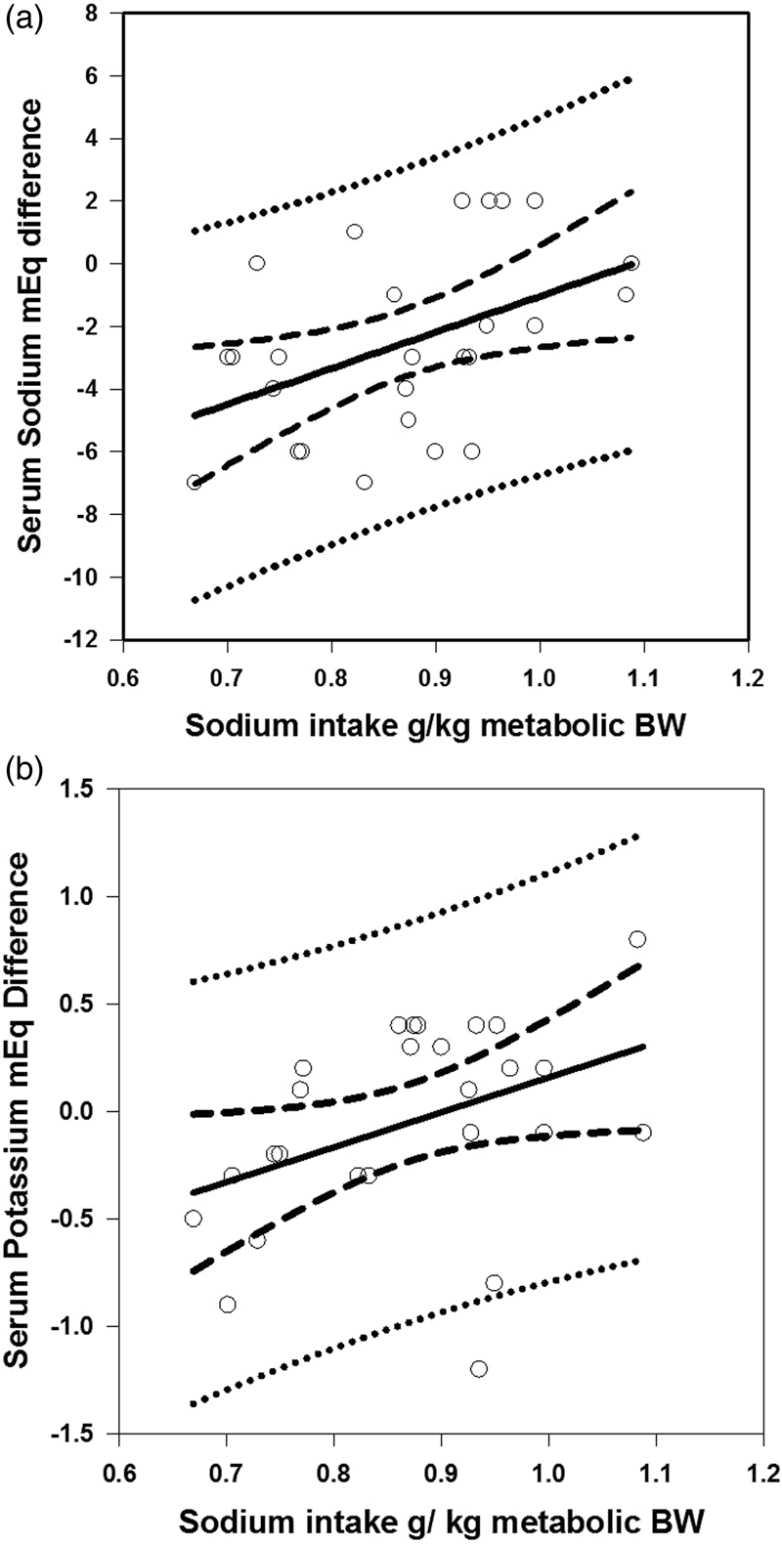
Linear regression analysis of sodium and potassium intake. (A) Linear regression of serum sodium difference between pre- and post-exercise and assumed dietary sodium intake per kg^0·75^. *R* = 0·45; *P* = 0·02. (B) Linear regression of serum potassium difference (mEq) between pre- and post-exercise and assumed dietary sodium intake per kg^0·75^. *R* = 0·40; *P* = 0·045. The solid line represents the linear regression; the dark dashed line represents the upper and lower 95 % mean confidence interval (CI); the light dashed line represents the upper and lower 95th percent individual CI.

Regression analysis of serum sodium difference from pre- to post-exercise in mEq of sodium compared with calculated sodium intake per kg metabolic body weight showed a significant correlation at *R* = 0·45 (*P* = 0·02; Fig. 1). Linear regression of potassium intake per kg metabolic body weight to mEq difference in serum potassium for each dog showed a significant negative correlation of *R* = −0·43 (*P* = 0·03), while the calculated linear regression analysis of sodium intake compared with mEq difference in potassium pre- to post-racing was modestly correlated and significant; *R* = 0·40, *P* = 0·045; Fig. 1). The slopes and intercepts of these two regressions were not significantly different. Mean differences and standard deviations in serum sodium between pre- and post-racing in team 1 = −0·1 (2·4) (*P* = 0·67); team 2 = −3·4 (2·7) (*P* < 0·01); and team 3 = −3·9 (2·4) (*P* < 0·01).

## Discussion

Like other endurance racing events we found a significant decrease of serum total protein, globulin and albumin^(^^2^^,^^4^^,^^5^^)^. These findings are thought to be due to increased protein catabolism due to the high-energy demand in these dogs, protein loss or potentially a state of chronic inflammation^(^^5^^)^. Markers of muscular damage increased due to racing as shown previously^(^^1^^,^^2^^,^^5^^,^^6^^)^. Our values are similar to other publications, but creatine kinase cannot be compared between different studies because of factors such as sex and age of dogs, distance travelled, and more importantly, inter-laboratory methods of analysis^(^^2^^,^^6^^)^. In addition, there were also rises in AST and ALT that were related to muscle permeability changes, rather than altered hepatic function^(^^2^^,^^17^^)^.

Few other reports have examined serum TAG, NEFA or ketone generation in endurance dogs. Others have shown variable serum TAG status^(^^2^^–^^4^^)^ and the present study shows a modest increase in serum TAG, while serum NEFA were markedly decreased post-exercise, which lends credence to the idea that fatty acid mobilisation is a significant component of exercise metabolism in these dogs. The energy requirement for thermoregulating and sustained prolonged exercise is enormous and the present data support the finding of McKenzie *et al.* who showed that muscle glycogenolysis is significantly reduced during the prolonged endurance exercise in sled dogs in favour of fatty acid metabolism^(^^18^^)^. This is further confirmed by the lack of ketone body generation observed in the present study which is similar to that observed by Kronfeld *et al.* whereby ketone generation during moderate high-intensity exercise in sled dogs is minimal when compared with human athletes^(^^19^^)^.

From an electrolyte perspective the most dramatic changes that occur in endurance sled dogs is mild hyponatraemia and hypokalaemia. These alterations rarely decrease below reference ranges; however, potassium decreases approach the lower end of the reference range in many studies^(^^1^^–^^4^^)^. The mild decrease in post-race serum values of sodium when compared with pre-racing values is not as dramatic as some studies from the 1990s examining similar long-distance races^(^^2^^–^^4^^)^. In those studies, the decrease of serum sodium varied from 2·9^(^^2^^)^ to 8·3 mEq/l^(^^3^^)^. We believe that the current diets adopted by most mushers are based on more commercial dog food than meat, and might better meet electrolyte requirements. Sodium intake appears to be related to serum sodium as the team supplemented with up to 3 g extra sodium per dog per d showed no alteration in serum sodium from pre- to post-race. To further support this we found that sodium intake was associated with the ability to maintain serum sodium concentrations across all dogs. Although sodium intake has not been reported in previous publications, it has long been assumed that sodium intake needs to be increased during endurance exercise, yet amounts have not been proposed. Mechanisms to maintain sodium homoeostasis in endurance sled dogs suggest hyperactivation of the renin–angiotensin–aldosterone system due to dramatically increased water turnover rate that leads to enhanced renal sodium excretion^(^^3^^)^. This coupled with higher renal solute load due to increased protein catabolism and urea filtration at the kidney leads to sodium loss in the face of heightened conservation mechanisms. Therefore, sodium intake should influence the serum status which we have shown.

Moreover, unlike other field studies we found no reduction in serum potassium, while other endurance races have shown decreases in potassium from 0·2 to 1 mEq/l^(^^2^^–^^4^^)^. Interestingly in the present study, potassium intake was negatively correlated with serum changes in potassium from pre- to post-racing, whereas sodium intake was positively correlated with serum potassium homoeostasis from pre- to post-racing. The present findings can be explained by higher sodium intake, particularly in team one, leading to less overall activation of renin–angiotensin–aldosterone system. Only one other study corroborates our findings^(^^5^^,^^18^^)^, whereby sled dog dogs were run 160 km/d for 5 d and received approximately 4·184 kJ/d in feed as a mix between commercial dog food and meat^(^^5^^,^^18^^,^^20^^)^. If this prior study were feeding similarly to what we have observed in the present study, then these dogs would have received between 7·5 and 10 g of sodium per d. Although speculative we hypothesise that the hyponatraemia and hypokalaemia may not be as prevalent as observed in previous studies due to increasing use of commercial dog food fed to endurance sled dogs, and that feeding over 1 g of sodium/4184 kJ may be warranted to prevent decreases in serum sodium and potassium.

Overall the present field study shows that the sodium and potassium changes in sled dogs are associated with dietary intake of sodium during racing. The present finding suggests that dietary sodium is highly conserved, but that this unique situation in the endurance canine athlete might warrant some divergence from the present recommendations for normal adult dogs to maintain metabolic equilibrium due to increased protein consumption and water turnover. Further well-controlled studies are essential to make firm recommendations regarding dietary needs for sodium intake due to endurance exercise, taking into consideration many factors, including energy and protein intake as well as water consumption and turn-over.
